# Application of circadian rhythm in osteoporosis prevention and treatment: from light, diet, and exercise to chronotherapy

**DOI:** 10.3389/fendo.2025.1698016

**Published:** 2025-12-01

**Authors:** Jun Qian, Kai Huang, Qiaocheng Zhai

**Affiliations:** 1 Division of Orthopaedics, The Quzhou Affiliated Hospital of Wenzhou Medical University, Quzhou People’s Hospital, Quzhou, China; 2 Orthopaedic Institute, Wuxi Ninth People’s Hospital Affiliated to Soochow University, Wuxi, China

**Keywords:** circadian clock, osteoporosis, chronotherapy, time-restricted feeding, exercise

## Abstract

Osteoporosis is a prevalent systemic bone disorder characterized by reduced bone mass, deterioration of bone microstructure, and increased bone fragility, significantly compromising the quality of life, particularly among elderly and postmenopausal populations. The circadian clock serves as a critical physiological mechanism for maintaining homeostasis and regulating rhythmic biological processes, playing an essential role in bone metabolism regulation. Recent studies have revealed strong associations between circadian disruption and the development and progression of osteoporosis, providing novel perspectives for intervention strategies. This comprehensive review examines the role of the circadian clock in osteoporosis and explores the current applications and future prospects of circadian-based interventions, including light exposure, dietary modifications, physical activity, and chronotherapy, in osteoporosis management. The objective is to offer comprehensive and precise strategic insights for osteoporosis prevention and treatment.

## 1 Introduction

The circadian clock is an evolutionarily conserved adaptation that enables organisms to align their internal physiology with daily environmental cycles. The central pacemaker, residing in the suprachiasmatic nucleus (SCN) of the hypothalamus, integrates photic cues and disseminates temporal signals via neural and humoral pathways to peripheral tissues. This coordination orchestrates a wide array of rhythmic physiological processes, including sleep-wake cycles, endocrine secretion, metabolic homeostasis, and other diurnal variations in bodily functions (1). At the molecular level, the core transcriptional regulators CLOCK and BMAL1 dimerize to form heterodimers that bind to E-box enhancer elements, thereby initiating the transcription of repressor encoding genes, such as Period (Per) and Cryptochrome (Cry). In parallel, the nuclear receptors REV-ERB and RORA contribute to the robustness and precision of circadian oscillations by exerting antagonistic effects on BMAL1/CLOCK transcriptional activity—through repression and activation, respectively (2).

Osteoporosis constitutes a major age-related chronic condition manifested through reduced bone mass and increased fracture susceptibility (3, 4). The condition develops when bone resorption mediated by osteoclasts exceeds bone formation regulated by osteoblasts. Notably, bone tissue demonstrates strong circadian regulation, with oscillating expression patterns observed in both core clock genes and bone turnover markers (5–9). Clinical observations have demonstrated that bone resorption markers (e.g., Type I collagen cross-linked C-terminal telopeptide, CTX) rise at night, a time when food intake is typically absent, and decline during daytime, which corresponds to periods of regular food intake, while bone formation markers (e.g., osteocalcin) are elevated during the resting phase (9–12). Furthermore, the suppression of CTX can be achieved by the intestinal-derived hormone glucagon-like peptide-2 (GLP-2), which is secreted during feeding (13–15). These findings collectively confirm the existence of precise circadian regulation of bone metabolic processes. Emerging evidence substantiates the circadian clock’s fundamental role in bone metabolism regulation. Genetic disruption of clock components in murine models disturbs osteoclast-osteoblast balance and alters bone metabolic phenotypes (16). Genetic ablation of *Bmal1* in osteoblasts and bone marrow mesenchymal stem cells (BMSCs) leads to increased expression of RANKL/OPG in cortical bone and elevated serum RANKL levels, thereby promoting osteoclast activation and subsequent bone loss (17, 18). Furthermore, studies have demonstrated that the circadian clock genes *Cry1* and *Bmal1* regulate osteogenic differentiation through mechanisms involving the β-catenin signaling pathway (19, 20). Additionally, *Bmal1* directly transcriptionally controls *Nfatc1* expression, thereby enhancing osteoclast differentiation. Consistent with this, conditional knockout of *Bmal1* in osteoclasts results in a significant increase in bone mass (21). Prenatal constant light exposure impairs tibial development in rat offspring, indicating long-term consequences of early-life circadian disruption on skeletal health (22). Clinical epidemiological studies demonstrate elevated osteoporosis and fracture risk among shift workers and individuals with sleep disorders (23–26). Furthermore, genetic association studies in elderly Chinese populations reveal significant osteoporosis risk modulation by polymorphisms in circadian genes (CRY2 rs2292910 and MTNR1B rs3781638), reinforcing the genetic involvement of circadian pathways in bone metabolism regulation (27).

Current osteoporosis management strategies encompass lifestyle interventions (nutritional supplementation, exercise programming, fall prevention), anti-resorptive agents (bisphosphonates, denosumab), bone-forming medications (teriparatide, romosozumab), and personalized treatment approaches (28). Nevertheless, clinical implementation of circadian principles in osteoporosis therapeutics remains limited. This review explores optimized intervention strategies through light-based therapies, temporally-restricted feeding, timed exercise interventions, and chronological drug administration for osteoporosis clinical management.

## 2 Light-based interventions

Light serves as the primary environmental cue for circadian entrainment. It also influences bone metabolism through vitamin D synthesis and melatonin secretion, making it a significant intervention for osteoporosis.

Solar ultraviolet B radiation catalyzes the conversion of 7-dehydrocholesterol in the skin to vitamin D3, which is subsequently hydroxylated in the liver and kidneys to form active 1,25-(OH)2D3. This metabolite promotes intestinal calcium absorption and regulates osteoblast differentiation, thereby supporting bone health (29, 30). In SAMP6 accelerated aging and OVX-induced osteoporosis murine models, exposure to 326 nm ultraviolet light elevated serum 25(OH)D levels and ameliorated skeletal deterioration (31, 32). Clinical studies demonstrate that UV irradiation over 24 weeks in vitamin D-deficient postmenopausal women significantly increased serum 25-(OH)D levels, enhanced femoral cortical thickness and bone strength, and reduced the bone resorption marker CTX, confirming that UV-mediated vitamin D synthesis can rectify imbalances in bone metabolism (32). Human studies have demonstrated that vitamin D levels in humans exhibit a distinct seasonal variation, with higher levels observed in summer and lower levels in winter. Meanwhile, the risk of fractures is significantly elevated during winter (33–36). Thus, vitamin D supplementation in winter is essential for preventing osteoporosis and reducing the risk of fractures.

Evidence indicates that individuals with unhealthy sleep patterns, including insomnia and excessive daytime sleepiness, have a significantly elevated risk for fractures (37). Shorter wavelengths, such as ultraviolet and blue light, exert more substantial effects on clock gene expression (38). Population studies indicate that morning blue light exposure improves sleep quality and activity rhythms, whereas evening blue light has detrimental effects on sleep and circadian alignment (39–41). Thus, morning blue light exposure could serve as a potential intervention to mitigate fracture risk through the improvement of sleep patterns.

Melatonin expression is subject to regulation by the circadian clock and environmental light (42, 43). Long-standing research indicates that melatonin plays a dual regulatory role in bone metabolism by stimulating osteogenesis and suppressing bone resorption, positioning it as a promising candidate for osteoporosis treatment (44–46). Artificial light at night (ALAN) is a major disruptor of circadian rhythms, adversely affecting bone health and suppressing pineal melatonin secretion. Clinical studies have shown that chronic nighttime light exposure in adults (e.g., shift work) elevates the bone resorption marker CTX and reduces the bone formation marker P1NP, disrupting bone metabolic balance and increasing osteoporosis risk (47). A comparative study revealed that children exhibit a markedly greater melatonin suppression in response to light exposure than adults. Specifically, 580 lux of light at night suppressed melatonin levels by 88.2% in children, relative to 46.3% in adults (48). Mitigation strategies include reducing exposure to blue light (450–495 nm) at night, ensuring 7–9 hours of darkness during sleep, and considering exogenous melatonin supplementation aligned with individual circadian rhythms to maintain circadian stability and bone metabolic equilibrium (44).

## 3 Dietary interventions

Dietary intake acts as a non-photic zeitgeber for peripheral clocks in tissues like bone and gut. The timing of food consumption influences bone metabolism, with Time-Restricted Feeding (TRF) being the most extensively studied temporal dietary pattern.

Time-Restricted Feeding confines daily food intake to a specific window without necessarily reducing caloric intake. Feeding misaligned with the active phase can desynchronize central and peripheral clocks, increasing metabolic burden (49). Conversely, TRF aligned with the active phase improves metabolic health (50–52). In animal studies, high-fat diet (HFD)-fed mice subjected to 10-hour TRF during their active phase showed reduced serum leptin levels and diminished inflammatory damage (e.g., synovial neutrophil infiltration, bone erosion), suggesting TRF may alleviate HFD-exacerbated bone damage by improving immune rhythms and suppressing inflammation (53). Concurrently, TRF reduced monocyte generation in obese mice by downregulating *Cebpa* expression in bone marrow hematopoietic stem/progenitor cells, maintaining immune cell homeostasis and indirectly protecting bone health (54). Thus, TRF may benefit bone health by modulating inflammation and immune homeostasis. In overweight humans, early TRF (e.g., 8:00 am to 2:00 pm) improved metabolic rhythms, enhanced autophagy, and exerted anti-aging effects compared to a longer eating window (8:00 am to 8:00 pm) (55), suggesting potential greater bone benefits from early active-phase TRF. However, 4-hour TRF during either the day or night did not affect bone quality in young 2-month-old mice, indicating that the protective effects of TRF might be more relevant under pathological conditions.

Clinical studies demonstrate that 12 weeks of 8-hour TRF in overweight adults significantly increased bone mineral content (BMC), confirming the bone-protective effects of TRF during weight loss (56). Food intake-associated GLP-2 can suppress the expression of CTX, a biomarker of bone resorption (13–15). Furthermore, GLP-2 has shown significant efficacy in ameliorating osteoporosis in both OVX and aged animal models of the disease (57–59). A 6-month randomized controlled trial found that while bone resorption marker CTX increased in the standard diet group among female university students with normal-weight obesity, it decreased in the 12-hour TRF group (60), indicating that TRF can also suppress CTX expression. Total body bone mineral content remained stable in the TRF group but significantly decreased in the standard diet group, further validating that TRF can mitigate weight loss-associated bone loss (60). However, an 8-week 8-hour TRF intervention in female university students with normal-weight obesity (BMI 18.5–23.9 kg/m², body fat percentage ≥30%) showed no significant effect on bone mineral density (61), suggesting minimal impact on bone metabolism in young, non-pathological populations. This study also noted that while TRF reduced BMI and body weight, it concurrently decreased lean tissue mass and increased total cholesterol, indicating the necessity for nutritional optimization (e.g., protein supplementation) in specific populations to avoid adverse effects (61).

## 4 Exercise interventions

Exercise is a potent stimulator of bone formation. Its timing can synergize with the circadian clock to optimize bone health benefits, with effects modulated by the timing, type, and intensity of exercise.

### 4.1 Timing of exercise

Animal studies suggest that exercise timing influences its osteogenic effects. Four-week-old mice that performed treadmill running (30 min/day, 5 days/week for 5 weeks) during the early active phase (as opposed to the late active or rest phase) exhibited significant increases in femoral length and upregulation of bone development-related genes (e.g., oxidative phosphorylation pathway genes). The rhythmic peak expression of oxidative phosphorylation genes in the chondrification center coincided with the early active phase, implying that exercise timing aligned with bone metabolic rhythms may yield synergistic benefits (62). However, in 12-month-old female rats, 2-hour treadmill running sessions during the rest phase (ZT4-6), early active phase (ZT12-14), or late active phase (ZT22-24) did not significantly differ in their effects on bone structural parameters (e.g., bone volume fraction BV/TV, connectivity density) or biomechanical properties, although all exercise groups showed elevated serum IGF-1 and irisin levels (63). This indicates that the sensitivity of bone to exercise timing may be age- and sex-dependent. However, meta-analysis found no significant differences between morning and evening resistance training regarding effects on muscle strength, hypertrophy, or bone density changes in human (64). Thus, a systematic understanding of how exercise type, intensity, and frequency affect femoral health across different age groups in human remains elusive. Although nocturnal rodents have an activity rhythm opposite to humans, the circadian rhythm of their serum bone turnover markers is consistent with that in humans (18, 65), characterized by a decrease during feeding or active phases. Therefore, it is hypothesized that early morning exercise may be more conducive to increasing bone mass in humans.

### 4.2 Synergistic effects of exercise type and intensity

High-impact exercises (e.g., jumping, running) and whole-body vibration (WBV), which generate significant mechanical load, demonstrate superior efficacy in improving bone mineral density compared to low-impact exercises. Studies show that high-impact exercise combined with WBV in postmenopausal women significantly increased lumbar spine and femoral neck BMD and reduced the bone resorption marker CTX, whereas low-impact activities like swimming showed no significant effect (66). Furthermore, postprandial exercise may enhance bone anabolism: 40 minutes of downhill walking (at supra-threshold speed to enhance momentum) 1 hour after a meal at 8:00 am in postmenopausal women significantly increased the osteogenic ratio (bone formation markers CICP, OC, BALP to bone resorption marker CTX) (67). Research by Borer et al. demonstrated that post-meal downhill locomotion (at 11:00 and 18:00), but not pre-meal exercise, enhances the osteogenic response (68). Besides, these investigations revealed that while uphill running elicits a sustained, high-level PTH release that promotes bone resorption and diminishes the osteogenic ratio, downhill running triggers only a brief PTH surge. This specific PTH profile is proposed to inhibit the nocturnal peak of CTX, thereby favoring a higher osteogenic ratio (67, 68). Collectively, while population-based studies have demonstrated that various types of exercise can promote bone health and have also indicated that the sequential order of exercise and eating behaviors influences the effect of exercise on bone metabolism, evidence from systematic studies investigating the temporal effects of different exercise types on bone health remains relatively scarce.

## 5 Chronotherapy of osteoporosis drugs

Chronotherapy involves optimizing drug administration timing based on circadian rhythms in drug metabolism and disease pathophysiology to enhance efficacy and minimize adverse effects. Its application in osteoporosis pharmacotherapy is well-substantiated. Current clinical chronotherapy for osteoporosis involves several pharmacological agents, including Teriparatide, Raloxifene, Salmon Calcitonin, and the potential drug candidate ONO-5334.

### 5.1 Bone-forming agents (Teriparatide)

Teriparatide (recombinant PTH1-34), a commonly used anabolic agent, exhibits efficacy influenced by administration timing. A 12-month randomized controlled trial demonstrated that postmenopausal osteoporotic women receiving morning injections of teriparatide achieved a significantly greater increase in lumbar spine BMD (9.1%) compared to the evening injection group (4.8%). The morning group also showed smaller increases in the bone formation marker PINP (215% vs. 358%) and the bone resorption marker TRAP5b (37% vs. 70%), suggesting morning administration promotes bone formation while avoiding excessive activation of bone resorption (69). Another study revealed that evening teriparatide injections do not alter the inherent circadian rhythm of CTX (which peaks nocturnally), while morning PTH administration significantly suppresses this rhythm and abolishes the nocturnal CTX peak (70). An ongoing randomized controlled trial (71) further comparing the effects of 08:00 versus 20:00 administration on bone turnover markers will provide more precise evidence for teriparatide chronotherapy.

### 5.2 Anti-resorptive agents

#### 5.2.1 Cathepsin K Inhibitor (ONO-5334)

ONO-5334 reduces bone resorption by inhibiting osteoclast cathepsin K activity. Studies indicate superior efficacy with morning administration. In healthy postmenopausal women, 5 days of ONO-5334 treatment resulted in a greater 24-hour area under the effect curve (AUEC) inhibition for serum CTX with morning dosing (69%) compared to evening dosing (63%). The AUEC inhibition for urinary CTX/Cr was also higher in the morning group (93% vs. 86%). This is mechanistically linked to higher plasma drug concentrations 12–24 hours after morning administration (trough concentration 9.4 ng/mL vs. 4.0 ng/mL), confirming morning as the optimal dosing time for ONO-5334 (72).

#### 5.2.2 Selective estrogen receptor modulator (raloxifene)

Raloxifene modulates bone metabolism via estrogen receptors but may increase venous thrombosis risk, necessitating dosing timing that balances efficacy and safety. In 39 postmenopausal osteoporotic women randomized to receive 60 mg/day raloxifene either in the morning or evening for 12 months, no significant differences were observed in changes of bone turnover markers (e.g., bone alkaline phosphatase, TRAP5b). However, plasma plasminogen activator inhibitor-1 (PAI-1), a marker associated with increased thrombosis risk, increased by 40.9% in the morning group but remained unchanged in the evening group, suggesting evening administration might be safer (73).

#### 5.2.3 Calcitonin (salmon calcitonin)

The efficacy of oral salmon calcitonin (sCT) is influenced by meal timing, with pre-meal administration being more effective. In healthy postmenopausal women, administration of 0.8 mg sCT at 08:00 (morning), 17:00 (pre-dinner), and 22:00 (evening) resulted in CTX inhibition rates of 75% for the pre-dinner and evening groups, significantly higher than the 40-50% inhibition in the morning group. This is likely due to higher bone resorption activity in the evening and avoided interference from food when administered pre-meal. Administration at 17:00 achieved 25% overall bone resorption inhibition, identifying it as the optimal dosing time (74).

## 6 Discussion

The circadian clock, through the synchronization of central and peripheral (bone) oscillators, precisely regulates bone metabolic rhythms. Circadian disruption (e.g., nighttime light exposure, shift work) constitutes a significant risk factor for osteoporosis. Circadian-based interventions demonstrate considerable potential: 1) Light: Specific wavelengths (UV, 680 nm) can improve bone health by promoting vitamin D synthesis and inhibiting osteoclast activity, while avoiding ALAN is crucial. 2) Diet: Active-phase TRF protects bone mass during weight loss, outperforming traditional dietary patterns. 3) Exercise: High-impact exercise during the early active phase, combined with postprandial timing, maximizes bone anabolic effects. 4) Pharmacotherapy: Chronotherapy optimizes treatment—morning administration for teriparatide and ONO-5334, evening administration for raloxifene, and pre-dinner for salmon calcitonin. However, the application of circadian rhythms in osteoporosis intervention is still in its nascent stage (**Table 1**).

**Table 1:** Clinical studies on circadian rhythm interventions for bone health.

Study population	Intervention method	Intervention effect	Reference
Overweight adults	12 weeks of 8-hour Time-Restricted Feeding (TRF)	Significantly increased Bone Mineral Content (BMC)	Lobene et al., 2021 (56)
Weight-loss responders (overweight/obese adults)	6 months of 12-hour Time-Restricted Eating (TRE)	Decreased bone resorption marker β-CTX; Total body bone mineral content remained stable (vs decrease in control)	Papageorgiou et al., 2023 (60)
Female university students with normal-weight obesity (high body fat %)	8 weeks of 8-hour Time-Restricted Feeding (TRF)	No significant effect on Bone Mineral Density (BMD)	Liu et al., 2023 (61)
Postmenopausal women	Morning injection of Teriparatide (vs. evening)	Significantly greater increase in lumbar spine BMD (9.1% vs 4.8%); Smaller increases in bone formation (PINP) and resorption (TRAP5b) markers	Michalska et al., 2012 (69)
Postmenopausal women	Morning vs. evening dosing of ONO-5334 (Cathepsin K inhibitor)	Morning dosing resulted in greater inhibition of serum and urinary CTX over 24 hours	Eastell et al., 2016 (72)
Postmenopausal women	Morning vs. evening dosing of Raloxifene (60 mg/day)	No significant difference in bone turnover markers; Morning dosing increased thrombotic risk marker PAI-1 by 40.9%	Ando et al., 2013 (73)
Postmenopausal women	Oral salmon calcitonin (0.8 mg) administered at 08:00, 17:00, or 22:00	CTX inhibition rates were higher with 17:00 (75%) and 22:00 (75%) dosing vs. 08:00 (40-50%)	Karsdal et al., 2008 (74)

Extensive evidence indicates that disruptions to the body’s biological rhythms—including those caused by aberrant light exposure, erratic eating patterns, and shift work—compromise bone health. Consequently, reinforcing these endogenous rhythms and identifying optimal timing for drug administration have emerged as crucial strategies in osteoporosis intervention. Multiple human studies demonstrate that morning light, particularly blue light, significantly suppresses melatonin secretion, elevates cortisol levels, and enhances daytime alertness (75–77). Thus, morning blue light exposure could strengthen the sleep-wake rhythm. During aging, the amplitude of circadian oscillations diminishes in multiple organs, the expression of circadian genes declines, and inter-organ communication weakens (78, 79). Specifically, *Bmal1* knockout in osteoblasts and bone marrow mesenchymal stem cells (BMSCs) activates osteoclasts and reduces bone mass (17, 18), suggesting that the attenuation of bone tissue rhythms during aging may contribute to osteoporosis. In mouse models with clock deficits, TRF can re-entrain hepatic rhythms (80). Furthermore, TRF aligned with the active phase synchronizes peripheral clocks and enhances communication among aging and metabolically disturbed organs (79, 81), which may partly explain its bone-improving effects. Similar to TRF, exercise acts as a Zeitgeber that reinforces the body’s circadian rhythms (82, 83). In mice, mechanical loading applied during the early active phase resulted in a greater endocortical bone formation response compared to loading during the inactive phase (5), indicating heightened bone sensitivity to anabolic stimuli at this time. Collectively, these findings suggest that early-active-phase exercise may prevent bone loss by both reinforcing systemic circadian rhythms and providing a more potent osteogenic stimulus. In humans, PTH secretion exhibits a biphasic circadian pattern: a moderate increase between 16:00 and 19:00, followed by a broader, more sustained rise from late evening to early morning, peaking between 02:00 and 06:00 (84, 85), while its levels remain low during the daytime. This nocturnal elevation pattern partially aligns with the rhythm of serum osteogenic markers. As an anabolic agent, morning administration of teriparatide produces a greater PTH surge and may therefore exert a more pronounced osteogenic effect than evening dosing. Conversely, most anti-resorptive medications (e.g., Raloxifene and Calcitonin) are optimally administered in the evening to target the period of heightened nocturnal bone resorption. Although morning dosing is recommended for ONO-5334, its peak effect occurs 12–24 hours post-administration (72), thereby coinciding with the night-time period of elevated bone resorptive activity. In conclusion, the timing of anti-osteoporotic drug administration should be carefully considered in the context of the inherent circadian rhythms of bone metabolism.

Future research should also prioritize the development of personalized chrono-interventions that leverage multi-omics approaches—such as genomics and metabolomics—coupled with data from wearable devices monitoring activity-rest patterns and light exposure, to design tailored strategies for specific populations, including postmenopausal women and shift workers. For example, shift workers may benefit from combined daytime light supplementation and fixed feeding windows. Additionally, exploring combined intervention strategies—such as time-restricted feeding (TRF), timed exercise, and chronotherapy—could yield synergistic benefits for bone health; a potential regimen might include 10-hour TRF, morning high-impact exercise, and morning teriparatide administration. Mechanistic studies should also be deepened through the application of single-cell sequencing, bone organoid models, and other advanced technologies to elucidate the intrinsic clock mechanisms regulating osteoblast and osteoclast functions, thereby facilitating the development of novel clock-targeting therapeutics.

In conclusion, circadian rhythm-based interventions represent a safe and sustainable strategic approach for osteoporosis. With ongoing research, they hold significant promise for becoming a cornerstone of precise osteoporosis prevention and treatment, offering a new paradigm for global bone health management.
